# Identification of F-Box/SPRY Domain-Containing Protein 1 (FBXO45) as a Prognostic Biomarker for TMPRSS2–ERG-Positive Primary Prostate Cancers

**DOI:** 10.3390/cancers15061890

**Published:** 2023-03-21

**Authors:** Marthe von Danwitz, Niklas Klümper, Marit Bernhardt, Alexander Cox, Philipp Krausewitz, Abdullah Alajati, Glen Kristiansen, Manuel Ritter, Jörg Ellinger, Johannes Stein

**Affiliations:** 1 Department of Urology, University Hospital Bonn, University of Bonn, 53127 Bonn, Germany; 2 Center for Integrated Oncology, University Hospital Bonn, University of Bonn, 53127 Bonn, Germany; 3 Institute of Experimental Oncology, University Hospital Bonn, University of Bonn, 53127 Bonn, Germany; 4 Institute of Pathology, University Hospital Bonn, University of Bonn, 53127 Bonn, Germany

**Keywords:** prostate cancer, FBXO45, prognostic biomarker, apoptosis

## Abstract

**Simple Summary:**

FBXO45 plays a role in the regulation of apoptosis through the ubiquitylation and degradation of specific target proteins. While FBXO45 has been suggested to have prognostic potential in various cancers, its specific role in prostate carcinoma (PCA) remains unclear. Transcriptome data concerning FBXO45 were analysed using The Cancer Genome Atlas (TCGA) and a publicly available Gene Expression Omnibus (GEO) progression PCA cohort. In addition, FBXO45 protein expression was evaluated using immunohistochemistry in a large cohort of PCA tissue microarrays. It was demonstrated that high FBXO45 expression was associated with advanced stages of PCA and biochemical recurrence. Shortened progression-free survival was associated with strong FBX045 staining, especially in TMPRSS2–ERG-positive PCA. In vitro experiments demonstrated that FBXO45 knockdown led to a significant reduction in migration capacity in the PC-3, DU-145 and LNCaP cell lines. These findings suggest that FBXO45 may serve as a promising biomarker for PCA and exhibit oncogenic properties.

**Abstract:**

Background: F-box/SPRY domain-containing protein 1 (FBXO45) plays a crucial role in the regulation of apoptosis via the ubiquitylation and degradation of specific targets. Recent studies indicate the prognostic potential of FBXO45 in several cancers. However, its specific role in prostate carcinoma remains unclear. Methods: A systematic analysis of FBXO45 mRNA expression in PCA was performed using The Cancer Genome Atlas database and a publicly available Gene Expression Omnibus progression PCA cohort. Subsequently, FBXO45 protein expression was assessed via immunohistochemical analysis of a comprehensive tissue microarray cohort. The expression data were correlated with the clinicopathological parameters and biochemical-free survival. The immunohistochemical analyses were stratified according to the TMPRSS2–ERG rearrangement status. To assess the impact of FBXO45 knockdown on the tumour proliferation capacity of cells and metastatic potential, transfection with antisense-oligonucleotides was conducted within a cell culture model. Results: FBXO45 mRNA expression was associated with adverse clinicopathological parameters in the TCGA cohort and was enhanced throughout progression to distant metastasis. FBXO45 was associated with shortened biochemical-free survival, which was pronounced for the TMPRSS2–ERG-positive tumours. In vitro, FBXO45 knockdown led to a significant reduction in migration capacity in the PC3, DU145 and LNCaP cell cultures. Conclusions: Comprehensive expression analysis and functional data suggest FBXO45 as a prognostic biomarker in PCA.

## 1. Introduction

Prostate cancer (PCA) is a common cancer in men and a major cause of cancer-related deaths globally [1]. While primary PCA can be potentially cured, the high rate of overtreatment highlights the limitations of current diagnostic tools, such as prostate-specific antigen (PSA) and grading according to the International Society of Urological Pathology (ISUP), in accurately discriminating between indolent and aggressive cases [2]. Despite significant advancements in medical therapy, mortality remains high and treatment options are limited for advanced stages of the disease [3,4]. A deeper understanding of PCA tumour biology and the identification of molecular biomarkers may improve risk assessment and treatment planning.

The current state of research supports the notion that PCAs have distinct molecular genetic signatures that require consideration in the process of identification of new biomarkers [5,6]. Thus, approximately half of all PCAs are characterised by chromosomal rearrangements leading to a chimeric oncogene through fusion of the promoter region of the androgen-induced transmembrane serine protease 2 (TMPRSS2) gene with the ETS-related gene (ERG) (TMPRSS2–ERG (T2E)). TMPRSS2–ERG fusion is recognised as a driver event for the progression to prostate cancer. Prior studies proved that T2E-positive and T2E-negative PCAs represent molecularly different PCA subtypes, and potential biomarkers should also be considered subtype specifically [6].

The Cancer Genome Atlas (TCGA) database allows us to identify potential biomarker candidates through an investigative approach. In a systematic analysis, we identified FBXO45 as an interesting prognostic gene and selected it for further comprehensive analysis. FBX045 is a member of the F-box proteins, which function as the substrate-specific adaptor subunit in ubiquitin ligase complexes that promote proteasomal degradation of critical cellular regulators [7,8,9,10]. It has already been reported that FBXO45 targets regulate cell apoptosis, neural development and carcinogenesis [11,12,13,14,15]. FBXO45 is a prognostic parameter in numerous cancers, such as squamous-cell lung carcinoma and gastric cancer [16,17,18].

However, the role of FBXO45 in PCA remains enigmatic. The present study aimed to investigate its potential role as a prognostic tissue-based biomarker for PCA.

## 2. Material and Methods

### 2.1. Transcriptome Data Assembly

The log2-transformed RNA sequencing data generated by IlluminaHiSeq and publicly available through the TCGA Research Network were downloaded via the UCSC Xena browser (http://xena.ucsc.edu accessed on 4 February 2023). The cohort comprised 497 primary PCA and 52 normal adjacent tissue (NAT) cases. The log-transformed, pre-normalised array signal intensities of the microarray data from a PCA progression cohort were downloaded via Gene Expression Omnibus (GEO, http://www.ncbi.nlm.nih.gov/geo/ accessed on 4 February 2023, GSE6919) [19]. The expression data of 25 androgen-deprivation-resistant metastatic samples derived from 4 patients were obtained from different metastatic sites and were thereby considered as individual samples (primary PCA (pPCA): *n* = 66, metastasis: *n* = 25).

### 2.2. Immunohistochemistry

In order to identify tissue-specific biomarkers that can facilitate more accurate prognostic predictions in the early stages following diagnosis, we chose to analyse radical prostatectomy specimens with comprehensive follow-up data, including biochemistry-free survival. Therefore, we immunohistochemically analysed the FBXO45 expression of a well-characterised primary PCA tissue microarray (TMA) cohort. The TMAs were constructed as described previously [20,21,22]. In brief, one to four tissue cores (1.2 mm diameter) of paraffin-embedded prostate tissue represented one case. Tumour samples with a lack of tissue or absence of carcinoma were excluded. In total, the tissue of 161 patients who underwent radical prostatectomy at the University Hospital of Bonn between 1998 and 2008 was available for analysis. The TMAs were freshly cut (3 µm) and mounted on Super Frost Plus Slides. Staining was performed using the polyclonal FBXO45-antibody (bs-13150R, dilution 1:300) on the Ventana Benchmark automated staining system following the manufacturer’s protocol (Ventana Medical System, Tucson, AZ, USA). The detection of the signal was performed using the ultraView Universal DAB detection kit. The slides were finally counterstained with haematoxylin and Bluing Reagent, dehydrated, mounted, and digitalised. The staining quality and specificity were confirmed by experienced uropathologists. Two observers independently rated the intensity of the FBXO45 staining as negative, weak, moderate, or strong (scores from 0 to 3). In the case of divergent rates, consensus was achieved by discussion. Each TMA core was assessed individually. If multiple TMA cores were available for one case, the mean value was calculated and considered for the further analyses. To investigate the prognostic role of FBXO45 expression, depending on the TMPRSS2–ERG status, a survival analysis regarding progression-free survival was performed based on both the total cohort and after stratification by TMPRSS2–ERG status (T2E-positive, *n* = 54 vs. T2E-negative, *n* = 107) as described by Gerke et al. [6]. Progression-free survival was defined as PSA-relapse-free (PSA ≥ 0.2 ng/mL) survival following radical prostatectomy.

### 2.3. Cell Lines and Culture Conditions

The human PCA cell lines (LNCaP, DU-145, PC-3, C4-2B) were obtained from the Leibniz Institute DSMZ—German Collection of Microorganisms and Cell Culture (Braunschweig, Germany). All the cell lines were cultured in RPMI1640 medium (Gibco; Thermo Fisher Scientific, Inc., Waltham, MA, USA) supplemented with 10% foetal bovine serum (FBS Superior, Biochrom GmbH, Berlin, Germany), 0.4% penicillin/streptomycin and 1% glutamine (Thermo Fisher Scientific, Inc., Darmstadt, Germany) in a humidified atmosphere of 5% CO_2_ and 95% air at 37 °C. The experiments were performed with mycoplasma-free and previously authenticated cell lines.

### 2.4. Antisense LNA GapmeR-Mediated Knockdown

We designed the specific FBXO45 GapmeR construct using the online available Antisense Gapmer Designer from Exiqon for the transient FBXO45 knockdown in vitro (Vedbaek, Denmark). The specific sequence of the construct was (5′A*G*C*G*C*G*C*A*G*A*A*G*A*G*A*T-3′). Furthermore, the following non-targeting negative control GapmeR sequence Negative Control A was used: (Neg Ctrl-A:) (5′-*C*G*T*A**G*T*C*G*A*G*G*A*A*G*T*A-3′). The transfections in the cell lines were performed in a final concentration of 150 nM at a ratio of 1:3 with the FuGENE HD-Transfection Reagent (#E2311, Promega Corporation, Madison, WI, USA) as defined in the manufacturer’s instructions.

### 2.5. RNA Isolation

Isolation of the RNA of the cell pellets was conducted using a Total RNA Purification Mini Spin Column Kit (Genaxxon bioscience GmbH, Ulm, DE, USA). After RNA isolation, the RNA quantity and quality were measured using a NanoDrop 2000 Spectrophotometer (Thermo Scientific, Wilmington, DE, USA).

### 2.6. Real-Time PCR

The mRNA expression of the corresponding cell lines was quantified 48 h post-transfection using RT-qPCR to analyse the knockdown efficiency. The following primer sequences were used: FBXO45 forward primer: 5′-GGCTGGAATCTGGTGGACAA-3′ and reverse primer: 5′-TCTCCTATCTGATATTTTGGTGCGT-3′; *β-actin* forward primer: 5′-CCAACCGCGAGAAGATGA-3′ and reverse primer: 5′-CCAGAGGCGTACAGGGATAG-3′. CDH1 forward primer: 5′-AAGGGGTCTGTCATGGAAGG-3′ and reverse primer: 5′-GGTGTTCACATCATCGTCCG-3′; CDH2 forward primer: 5′-CCATCATTGCCATCCTGCTC-3′ and reverse primer: 5′-GTTTGGCCTGGCGTTCTTTA-3′; SNAI1 forward primer: 5′-GCTGCAGGACTCTAATCCAGA-3′ and reverse primer: 5′-ATCTCCGGAGGTGGGATG-3′; SNAI2 forward primer: 5′-TGGTTGCTTCAAGGACACAT-3′ and reverse primer: 5′-GTTGCAGTGAGGGCAAGAA-3′; ZEB1 forward primer: 5′-CAGGGAGGAGCAGTGAAAGA-3′ and reverse primer: 5′-ACATCCTGCTTCATCTGCCT-3′; and VIM forward primer: 5′-GAGAGGAAGCCGAAAACACC-3′ and reverse primer: 5′-TTGCGTTCAAGGTCAAGACG-3′.

The different levels of mRNA expression were standardised to *β-actin* and quantified using the ΔΔ CT method.

### 2.7. Western Blot

Western blots were used to validate the FBXO45 knockdown efficiency at the translational level in the transfected PCA cell lines: After 72 h of transfection, cells were collected and homogenised in 100–200 µL RIPA cell lysis buffer (300 mM NaCl 5 M, 20 mM Tris 500 mM pH 7.5, 2% Nonidet^®^ P40 BioChemica, Espoo, Finland, A1694 AppliChem Panreac, Darmstadt, Germany, 0.5% sodium deoxycholate D6750 Sigma-Aldrich, 0.2% SDS (*sodium dodecyl sulphate*) 10% L3771 Sigma-Aldrich, St. Louis, MO, USA, 70% H_2_O, Ampuwa), including a protease inhibitor cocktail (Complete Mini EDTA-free, Roche, Basel, Switzerland, 10 mM NaF (*sodium fluoride*) 200 mM S7920 Sigma-Aldrich). The protein concentration was determined with a BCA protein assay kit (Thermo Fisher Scientific, Inc., Waltham, MA, USA).

The primary antibody against FBXO45 was used for incubation at a dilution of 1:500 (LS-C169575, Rabbit, LSBio, Seattle, WA, USA). As an internal loading control antibody, anti-alpha-tubulin was used at a dilution of 1:4000 (#A5316, mouse, Sigma-Aldrich). We performed the signal detection with horseradish peroxidase-associated antibodies (antirabbit POD, #7074, Cell Signaling, and anti-mouse-POD, #170-6516, Bio-Rad, Hercules, CA, USA). To visualise the chemiluminescence signal, we used a SuperSignal WestFemto Kit (Thermo Scientific, Waltham, MA, USA) and an LAS 3000 Image Reader (Fujifilm, Tokyo, Japan) for documentation. Densitometry was performed using the software ImageJ as described previously by Stael et al. [23].

### 2.8. Cell Proliferation Assays

An EZ4U cell proliferation assay kit was used according to the manufacturer’s protocol (EZ4U; Biomedica Group, Vienna, Austria). A total of 2000 PC3 and DU-145 cells/well or 3000 LNCaP cells/well were seeded in 96-well plates in 100 µL medium and incubated overnight. The GapmeR transfections were then conducted with the adhered cells. We measured the 96-well plates 48 h and 72 h post-transfection by quantifying the colour absorbance spectrometrically in a microplate reader (Tecan Spectra Thermo, SLT Labinstruments Deutschland GmbH) at a 450 nm wavelength, with a 620 nm reference. We conducted the experiments in triplicates.

### 2.9. Migration Assays

After a 48 h period following transfection, we harvested the cells and seeded them into migration Boyden chambers. A total of 5 × 10^4^ cells of PC-3 and 1.5 × 10^5^ cells of DU-145 and LNCaP were plated in the upper chamber of the migration inserts (VWR, Darmstadt, Germany) containing RPMI medium without FCS. For the chemotactic attraction, we filled a medium containing 10% FCS in the lower chamber. The cells were fixed after 48 h with 4% formaldehyde (PanReac AppliChem, Darmstadt, Germany), washed and permeabilisated with TBST (50 mM Tris, 150 mM NaCl, 0.05% Tween-20, pH 7.5), and then coloured with haematoxylin (Vector Hematoxylin Counterstain H-3404, Vector Laboratories) and washed with water three times. We scanned the membranes and the cell number was determined automatically via nucleus detection using the QuPath software. Each experiment was conducted three times.

### 2.10. Statistical Analysis

Microsoft Excel, SPSS, and GraphPad Prism were used for the statistical analyses and visualisation of the data. The non-parametric Mann–Whitney U test was used for the group comparisons. The non-parametric Kruskal–Wallis test was applied to test three or more groups. Survival analyses were performed using the Kaplan–Meier estimate curves and log-rank tests. Thus, multivariate Cox regression analyses were performed after the co-adjustment of the TNM stage, ISUP groups and age to provide an independent and additive prognostic value for the patients’ progression-free survival.

### 2.11. Ethical Approval and Consent to Participate

All the patients gave written informed consent for the collection of biomaterials. The study was approved by the ethics committee of the University Hospital Bonn (number: 013/20).

## 3. Results

### 3.1. F-Box/SPRY Domain-Containing Protein 1 (FBXO45) In Silico Expression Analysis

**Figure 1:**
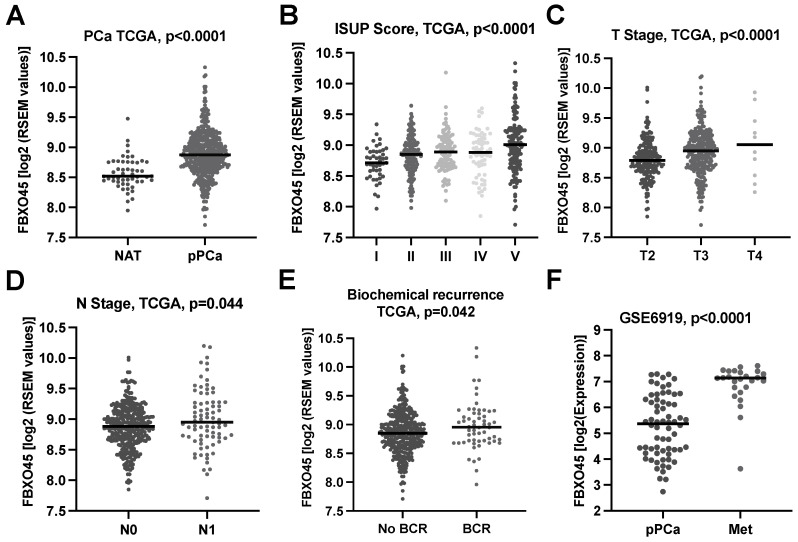
FBXO45 expression is enhanced in aggressive primary prostate cancers (pPCA) and distant metastasis (Met). (**A**–**E**) are based on the PCA TCGA dataset: (**A**) Distribution of FBXO45 RNA expression in normal adjacent prostatic tissue (NAT) in comparison to primary prostate cancer (pPCA). (**B**) The scatter plots depict FBXO45 RNA expression across ISUP subgroups (**B**), by T Stage (**C**), by N Stage (**D**) and by biochemical recurrence status (BCR) in (**E**). (**F**) Illustration of FBXO45 RNA expression in pPCA compared to distant metastasis (Met) in the PCA progression cohort GSE6919.

To investigate the impact of FBXO45 in PCA, we correlated the FBOX045 expression with the clinicopathological parameters and the clinical course of the patients using the PCA TCGA dataset (*n* = 497). The analysis revealed significantly higher FBXO45 expression in pPCA compared to NAT (*p* < 0.0001, Figure 1A). In addition, the analysis showed the significant association of the FBXO45 expression with the adverse clinicopathological parameters ISUP grading groups (*p* < 0.0001, Figure 1B), pT stage (*p* < 0.0001, Figure 1C), and pN stage (*p* = 0.044, Figure 1D). Furthermore, high FBXO45 expression was associated with biochemical recurrence (*p* = 0.042, Figure 1E). To further elucidate whether the FBX045 expression varies in primary tumours and metastasis, we analysed the PCA progression cohort GSE6919. This analysis revealed significantly increased FBXO45 RNA expression throughout the progression to distant metastasis (Met) (*p* < 0.0001, Figure 1F).

### 3.2. FBXO45 Protein Expression on a PCA Tissue Microarray (TMA)

**Figure 2:**
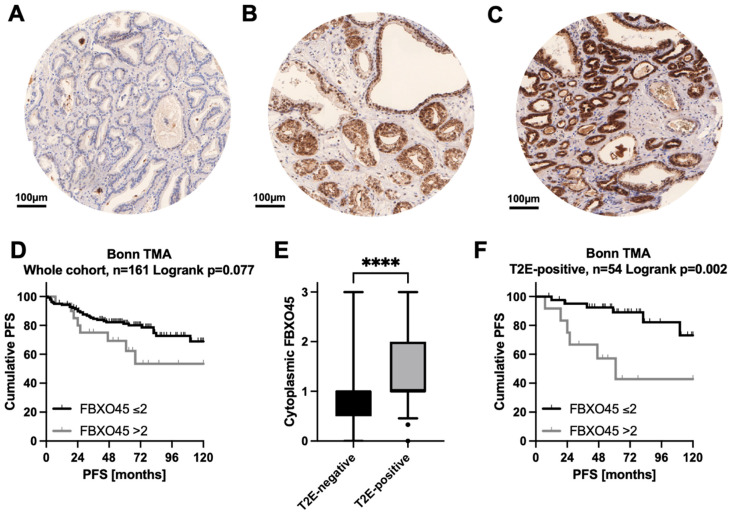
Increased cytoplasmic FBXO45 expression is associated with unfavourable progression-free survival (PFS) in the TMRPSS2–ERG (T2E)-positive primary prostate cancer (pPCA) subgroup. (**A**–**C**) Representative immunohistochemical staining for FBXO45 on primary prostate cancers (pPCA) at 10× magnification, scale bars = 100 μm. (**A**) Absence of FBXO45 expression in pPCA. Moderate (**B**) and strong (**C**) cytoplasmic FBXO45 expression in pPCAa. (**D**) Kaplan–Meier curve using the FBXO45 cut-off 2 (negative to moderate versus strong cytoplasmic FBXO45 protein expression) with regard to PFS. (**E**) Boxplot depicting cytoplasmic FBXO45 expression for the TMPRSS2–ERG (T2E)-negative (*n* = 107) versus -positive (*n* = 54) subgroup. (**F**) Strong cytoplasmic FBXO45 expression is associated with unfavourable PFS in the T2E-positive subgroup. **** *p* ≤ 0.0001.

To further ascertain the prognostic impact of FBXO45 at the protein level, we immunohistochemically stained and evaluated a large PCA-TMA cohort (*n* = 161). The immunohistochemical analysis revealed the predominantly cytoplasmic staining of the carcinoma cells. A total of 12.1% of the cases were FBXO45-negative (no staining, intensity 0), 57.8% were weakly stained (0 < mean staining intensity ≤ 1), 18.5% were moderately stained (1 < mean staining intensity ≤ 2) and 11.6% were strongly stained (mean staining intensity > 2, see Figure 2A–C for representative staining examples). The Kaplan–Meier analysis showed a modest trend (*p* = 0.077) regarding the association of strong FBXO45 staining (mean staining intensity > 2) and shortened PSA-free survival after prostatectomy. In an exploratory analysis of the Bonn TMA cohort, we found that the cytoplasmic FBXO45 protein expression was significantly higher in the TMPRSS2–ERG (T2E)-positive subgroup (Figure 2E). Kaplan Meier analysis of the overall cohort showed an association of strong FBXO45 staining with a shortened PFS in terms of modest trend (log-rank *p* = 0.077, Figure 2D). Of note, strong cytoplasmic FBXO45 protein expression was significantly associated with shortened PFS in the T2E-positive subgroup (Figure 2F), but not in the T2E-negative subgroup (log-rank *p*-value for T2E-negative = 0.893). Within the T2E-positive subgroup, the multivariate Cox regression analysis confirmed strong cytoplasmic FBXO45 (mean staining intensity > 2) as an independent prognostic parameter (hazard ratio 4.483, *p* = 0.022) when co-adjusted for the pT stage, pN stage, ISUP groups and age (Table 1).

### 3.3. Functional Characterization of FBXO45 In Vitro

**Figure 3:**
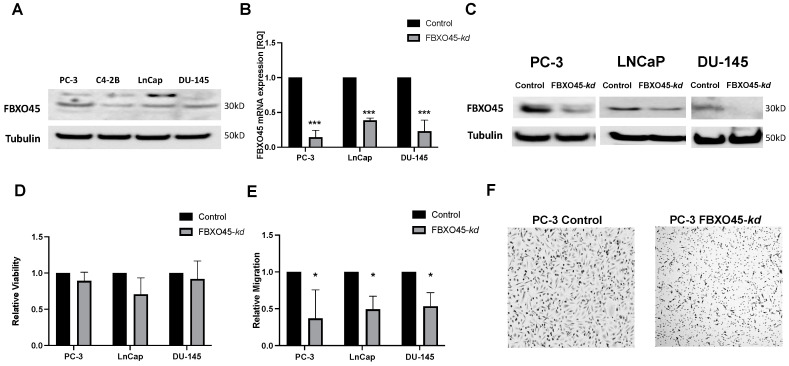
Functional impact of FBXO45 loss in prostate cancer cell culture models. (**A**) Baseline FBXO45 protein expression in different prostate cancer cell lines. (**B**) FBXO45 mRNA (**B**) and protein (**C**) expression following Antisense LNA GapmeR-mediated FBXO45-knockdown (FBXO45-*kd*). (**D**) Impact of FBXO45-*kd* on relative viability in our prostate cancer cell lines compared to the control. (**E**) FBXO45-*kd* impairs migration capacity according to the Boyden chamber assay; representative images of the Boyden chamber membrane depicted for PC-3 (**F**). Experiments were performed in triplicates. * *p* ≤ 0.05; *** *p* ≤ 0.001.

To further elucidate the functional role of FBXO45 in vitro, we aimed to induce transient FBXO45 knockdown (FBXO45-kd) using the established antisense locked nuclear acid (LNA)/GapmeR-mediated system on several established PCA cell culture models. First, we screened the cell lines PC-3, C4-2B, LNCaP and DU-145 for their FBXO45 protein expression under standard conditions. Since PC-3, LNCaP and DU-145 showed the strongest protein expression of FBXO45, we chose them for the subsequent functional analyses (Figure 3A). We were able to establish efficient FBXO45-kd for PC-3, LNCaP and DU-145, which was confirmed at both the mRNA level and the protein level, detected by RT-qPCR (Figure 3B), and confirmed by Western blot (Figure 3C). The densitometry data and uncropped Western blots are available in the Supplementary Materials. To subsequently investigate the functional impact of FBXO45 knockdown on tumour behaviour, we first examined the effect of FBXO45 on cell growth in a proliferation and cytotoxicity assay. The proliferation capacity of the tumour cells was not changed (Figure 3D). However, FBXO45 depletion strongly decreased the migration capacity of the PCA cells according to the Boyden chamber assays (Figure 3E or Figure 3F). In summary, we hypothesise that FBXO45 expression in PCA cell culture models is essential for maintaining oncogenic traits and specific FBXO45 knockdown reduces the ability for metastatic spread.

## 4. Discussion

In this study, we investigated the prognostic role of FBXO45 in PCA and its functional role in metastatic disease progression. Several lines of evidence support the notion that the gene has oncogenic properties in PCA and could, therefore, be a promising candidate for a new biomarker.

First, we demonstrated that FBXO45 is overexpressed and is associated with adverse clinicopathological parameters and shortened progression-free survival based on an analysis of a large mRNA cohort. Second, the investigation of a PCA progression cohort revealed a further upregulation of FBXO45 in PCA metastasis. These results were supported by an analysis of FBXO45 at the protein level. Remarkably, the principle of considering different molecular subtypes in biomarker studies was supported in this study. As described by Gerke et al. [6], in approximately 50% of PCA cases, a chromosomal rearrangement that forms a chimeric oncogene through fusion of TMPRSS2 with ERG (TMPRSS2:ERG or T2E) is present, which may exploit different gene signatures or pathways to promote the malignancy of PCA [6,24,25]. In recent years, TMPRSS2:ERG has been a major focus of research in the field of prostate cancer [6,25,26]. It has been shown that ERG overexpression is mostly related to the gene fusion of TMPRSS2 and ERG [27]. PCA is characterised by the fusion of the promoter region of TMPRSS2 with the coding region of ERG. The promoter region of TMPRSS2 is known to contain androgen-sensitive elements, which allows the overexpression of ERG to be driven by androgens [27,28]. Currently, this gene fusion is considered to be a driver event for carcinogenesis in prostate cancer [26]. TMPRSS2:ERG fusion is known to play a critical role in driving the progression from prostatic intraepithelial neoplasia (PIN) to invasive carcinoma [29]. Normal prostate tissue does not express ERG under regular circumstances [30]. The overexpression of ERG leads to the dysregulation of cell proliferation, differentiation, angiogenesis, inflammation and apoptosis, and it is thereby considered one of the main factors for the transformation from localised to aggressive and metastatic cancer [26,31]. Nevertheless, only 50% of PCAs are T2E-positive. Gerke et al. [6] showed that PCAs should be classified as different molecular subtypes based on their T2E status. Overall, their study demonstrated that the T2E status, which itself is not a strong prognostic biomarker, critically determines the prognostic value of other biomarkers. They identified five prognostic biomarkers associated with a worse outcome exclusively in T2E-negative PCA. Remarkably, no biomarker was found in the T2E-positive subgroup. The authors concluded that the molecular subtype must be considered when applying prognostic biomarkers for outcome prediction in PCA [6]. Therefore, we aimed to assess a subtype-specific analysis based on the T2E-negative and T2E-positive molecular subtypes in this study. The present work revealed a T2E-positive status in approximately 1/3 of cases (54/161), which is slightly lower than the published data [32]. FBXO45 is significantly overexpressed in T2E-positive PCA and is also associated with an unfavourable clinical course only in this subgroup, which further supports the subtype-specific consideration, especially concerning TMRPSS2–ERG fusion, in the development of robust prognostic biomarker models for PCA. Remarkably, this is the first biomarker—to the best of our knowledge—that is identified in the T2E-positive subgroup. Finally, functional analyses in vitro revealed that FBXO45 has an impact on the cell motility and migration capacity in three metastatic PCA cell lines, suggesting that the metastatic ability of PCA is at least in part influenced by FBXO45 activity.

Placing the results in a functional context, FBXO45 represents the substrate-recognition subunit of the E3 ligases that control protein degradation via ubiquitylation. Recent scientific works demonstrated its pivotal role in numerous human diseases, neuronal development, tumorigenesis and tumour progression. It was shown that FBXO45 is highly expressed in gastric cancer and squamous-cell lung carcinoma and correlated with shortened survival [16,17]. Furthermore, there is evidence that FBXO45 facilitates tumour progression in pancreatic cancer [33]. The unfavourable prognostic role in tumour diseases is attributed to the anti-apoptotic function of FBXO45, resulting from the various target proteins that predominantly act as tumour suppressors. Thus, FBXO45 ubiquitinates and degrades the target p73, a member of the p53 family, leading to a reduction in cell death [12]. Another target that has already been investigated is the prostate apoptosis response protein 4 (Par-4). Par-4 is a tumour suppressor that induces cancer-selective apoptosis. Loss or downregulation of Par-4 has already been shown to be associated with therapy resistance and tumour relapse [11,18,34]. Another interesting target of FBXO45, FBXW7, is a tumour suppressor that targets specific substrates for ubiquitination and degradation, including the known driver oncogenes of aggressive PCA MYC and Mcl-1 [18,35,36]. Remarkably, FBX045—mediated by FBXW7—decreases sensitivity to spindle poisons such as taxanes [37]. From a therapeutical point of view, this is of particular interest since docetaxel and cabazitaxel are used as chemotherapy agents for metastatic PCA.

In the last few years, microRNAs have increasingly gained attention in uro-oncological research. FBX045 might also play a role in this context. A recent study showed that FBXO45 is downregulated by the tumour-suppressive microRNA-30e [38]. Overexpression of FBX045 might be driven by the downregulation of microRNA-30e in PCA. Vice versa, the anticancer function of microRNA-30e could be mediated by the downregulation of FBX045, suggesting a possible regulatory mechanism.

## 5. Conclusions

In a synopsis of the literature and the results of this work, FBXO45 overexpression seems to mediate relevant oncogenic properties in PCA and represent an independent prognostic biomarker. The functional data also suggest that FBXO45 is involved in the formation of metastasis. Of particular clinical relevance is the potential impact of FBX045 on the tumour response to taxane-based chemotherapy. Furthermore, our work strengthens the classification of PCA into different molecular subtypes. Implementation of molecular subtyping in clinical practice could enable accurate prognostic predictions and pave the way towards individualised therapy.

## Figures and Tables

**Table 1:** Multivariate Cox regression analyses of the T2E-positive subgroup (TMA cohort) regarding progression-free survival (PFS).

Parameters	*p*-Value	Hazard Ratio	95% Confidence Interval	
			Low	High
FBX045	0.022	4.483	1.246	16.136
pT	0.372	1.943	0.452	8.357
pN	0.471	0.404	0.034	4.751
ISUP	0.636	0.972	0.866	1.092
Age	0.636	0.972	0.866	1.092

## Data Availability

The data presented in this study are available on request from the corresponding author.

## Supplementary Materials

The following supporting information can be downloaded at: https://www.mdpi.com/article/10.3390/cancers15061890/s1, Table S1. Supplemental data_Table_Western_Blot_Densitometry, Figure S1. Supplemental_Figure_Western_Blot.
