# Supplementary figures and images for: Identification of F-Box/SPRY Domain-Containing Protein 1 (FBXO45) as a Prognostic Biomarker for TMPRSS2–ERG-Positive Primary Prostate Cancers
Source: Cancers (Basel). 2023 Mar 21;15(6):1890. doi: 10.3390/cancers15061890 (PMC10046786; doi:10.3390/cancers15061890)

## Screening

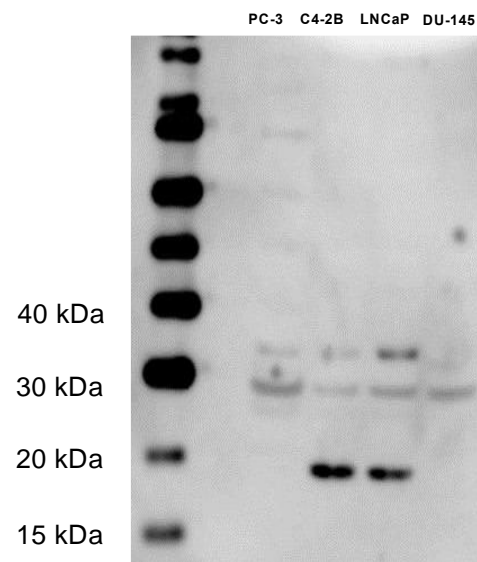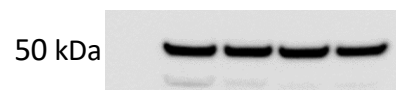

## LNCaP

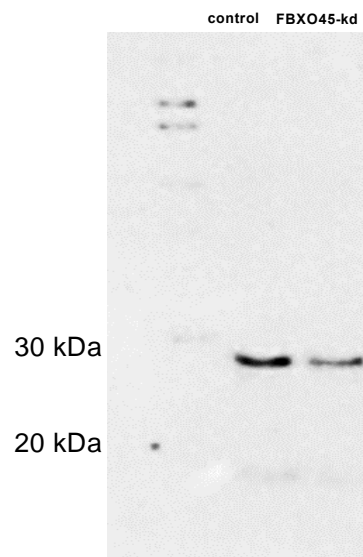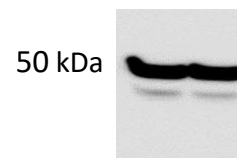

## PC-3

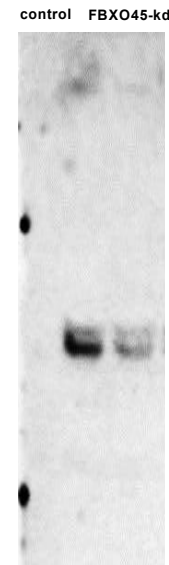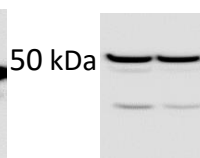

## DU-145

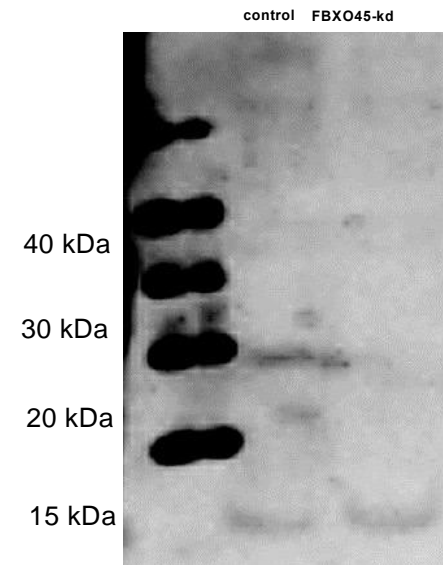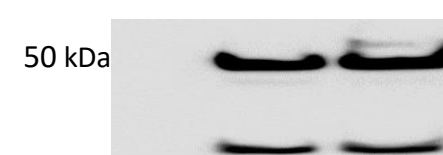

Supplement: Supplementary file 1 [file cancers-15-01890-s001.zip › Figure S1. Supplemental_Figure_Western_Blot.pdf]
